# Workflow for efficiently isolating microspore cultures of different rice genotypes by optimizing the callus induction medium

**DOI:** 10.3389/fpls.2025.1662463

**Published:** 2025-09-30

**Authors:** Zhiwei Chen, Guimei Guo, Shuwei Zhang, Ting He, Shiji Feng, Chenghong Liu, Yu Wang, Longhua Zhou

**Affiliations:** Shanghai Key Laboratory of Agricultural Genetics and Breeding, Biotechnology Research Institute, Shanghai Academy of Agricultural Sciences, Shanghai, China

**Keywords:** *Oryza sativa* L., microspore culture, callus induction, plant regeneration, ploidy identification

## Abstract

As one of the most important staple foods in the world, rice plays a key role in global food security. Doubled haploid technology based on isolated microspore culture can shorten the time taken for rice breeding programs. However, this technology still faces many problems, such as genotypic dependency, low culture efficiency, and a shortage of skilled workers. In this study, 15 rice genotypes, comprising 12 *japonica* genotypes and 3 *indica* genotypes, were randomly selected for microspore culture research, and the effects of different callus induction media (CIMs) on callus induction were compared and the related plant regenerations were also shown. The results showed that maltose was the optimal carbon source and the CIM III was the best for callus induction by comparing the number of rice genotypes that could be induced to form calli and the callus yields. For plant differentiation, 12 of the 14 rice genotypes regenerated green seedlings, all of which were *japonica* rice genotypes. Ploidy identification showed that the spontaneous doubling rate of regenerated seedlings from isolated microspore cultures ranged from 14.3 to 98%, which was higher than that observed in anther cultures. In conclusion, this study established an isolated microspore culture method that is suitable for different rice genotypes, providing more options for using doubled haploid technology in rice.

## Introduction

Haploid breeding technology, also known as doubled haploid (DH) breeding technology, can obtain homozygous recombinants in 1 or 2 generations, shortening the process for breeding new crop varieties; thus, breeders are interested in this technology (Hale et al., 2022). Rice, one of the most important food crops in the world and a staple food in Asian countries, is consumed by more than half of the world’s population, making it crucial for ensuring global food security (Wei et al., 2024). Therefore, carrying out research on DH technology in rice is of great significance.

Since Chen et al. (1980) first reported isolated microspore cultures in rice in the last century, research progress using this technology in rice has been relatively slow, with only sporadic reports (Raina and Irfan, 1998; Islam et al., 2013; Rahman et al., 2022). However, isolated microspore cultures have been reported to be more efficient than anther cultures (Li and Devaux, 2005), and this method can exclude the interference of somatic tissues, such as the epidermis, middle layer, tapetum, and connective tissue of the anther. Moreover, calli from mixed microspores obtained via isolated microspore culture are more representative for use in molecular mechanism studies than those from individual anthers obtained via anther culture. Therefore, the establishment of an efficient isolated microspore culture technology in rice can accelerate the development of DH breeding and be better used for analyzing molecular mechanisms relevant to microspore culture of rice.

In previous studies, an efficient isolated microspore culture technology in barley was established by Lu et al. (2016) and Chen et al. (2023), and an efficient anther culture technology in rice was also established recently (Guo et al., 2024) and the key steps involved in isolated microspore culture in rice was initially explored (Gao et al., 2024). In this study, combined with the results of previous research, the callus induction step was further optimized, and an efficient microspore culture technology suitable for different rice genotypes was established, providing feasible solutions for the popularization and large-scale use of this technology.

## Materials and methods

### Plant materials

A total of 15 rice genotypes that were previously used for anther culture (Guo et al., 2024) were used in the present study. Among them, 11 were obtained from the National Mid-term Bank for Rice (Fuyang District, Hangzhou City, Zhejiang Province, China), and the remaining 4 were obtained from the Crop Breeding and Cultivation Research Institute of the Shanghai Academy of Agricultural Sciences (Fengxian District, Shanghai City, China) (**Table 1**). The seeds of these rice genotypes were sown in the seedling field at the Chonggu Experimental Base of the Shanghai Academy of Agricultural Sciences (Qingpu District, Shanghai City, China), and seedlings were transplanted to the paddy fields.

**Table 1:** Different rice genotypes used in this study.

Rice genotype	Code	Subspecies	Origin
Nipponbare	Q1	*Japonica*	National Mid-term Bank for Rice, China
Wuyunjing 7	Q2	*Japonica*	
93-11	Q3	*Indica*	
Zhonghua 11	Q4	*Japonica*	
Nanjing 46	Q5	*Japonica*	
Zhongzao 39	Q6	*Indica*	
Zhongjiazao 17	Q7	*Indica*	
Xiushui 134	Q8	*Japonica*	
Nanjing 9108	Q9	*Japonica*	
Nanjing 5055	Q10	*Japonica*	
Shangshida 19	Q11	*Japonica*	
7375	Q12	*Japonica*	Crop Breeding & Cultivation Research Institute of SAAS
Hudao 89	Q13	*Japonica*	
Huruan 1212kang	Q14	*Japonica*	
Huxiangruan 450	Q15	*Japonica*	

### Tiller collection and low-temperature pretreatment

**Figure 1:**
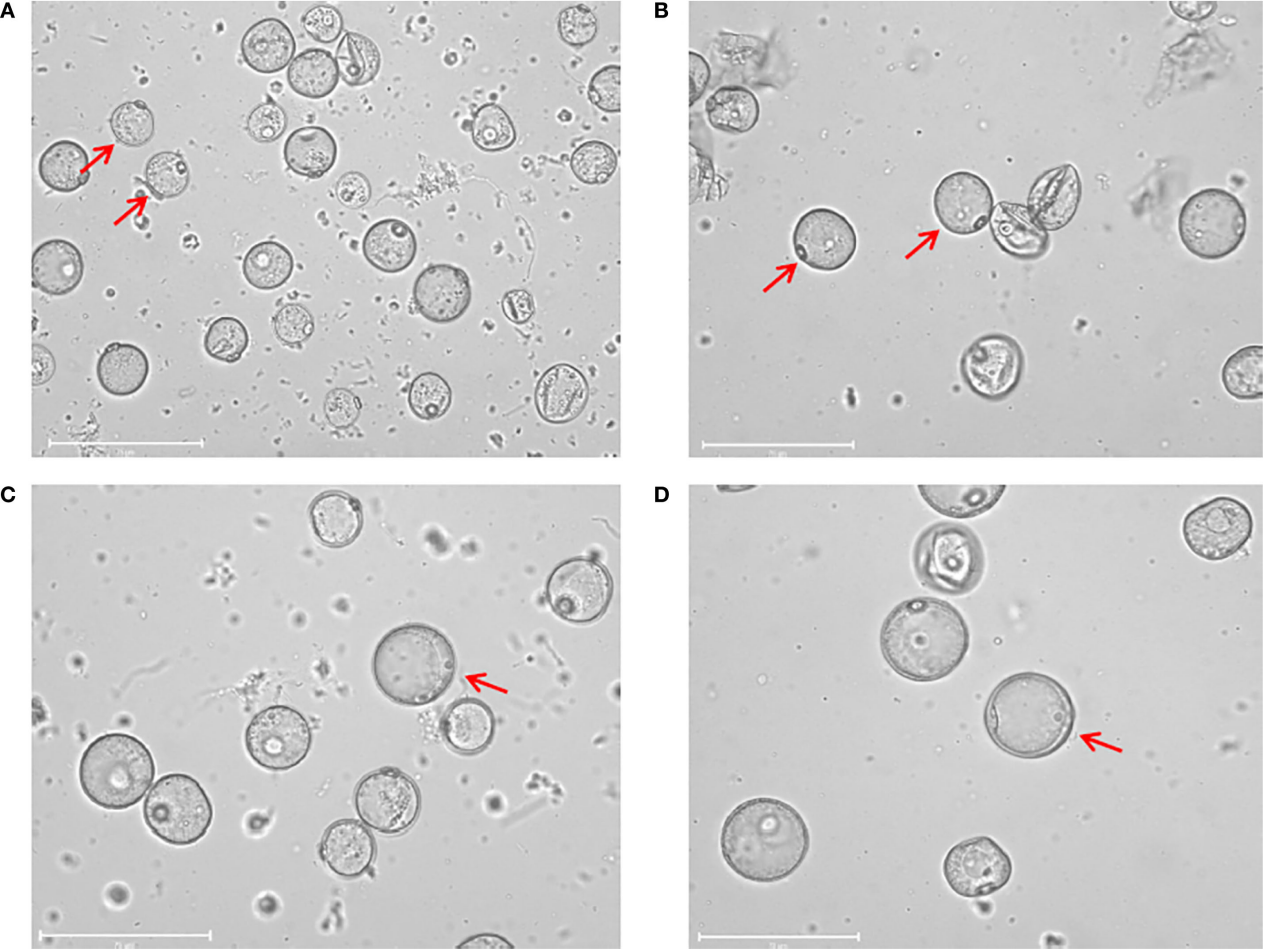
Isolated microspores at different developmental stage. **(A)** Microspores at the early uni-nucleate stage. **(B)** Microspores at the middle uni-nucleate stage. **(C)** Microspores at the late uni-nucleate stage. **(D)** Microspores at the early bi-nucleate stage. Scale bar=75 μm.

Tillers in the booting stage were collected according to the method described by Guo et al. (2024). During the booting stage, healthy tillers of each rice genotype were collected between 8 and 10a.m. after ensuring that the microspore development stage in the florets from the middle of the panicles was at the late uni-nucleate to early bi-nucleate stage (**Figure 1**). The tillers collected from each rice genotype were trimmed neatly, wrapped in wet gauze, and sealed in polyethylene bags to prevent dehydration. However, this method required a longer low-temperature treatment duration than that for anther culture (5°C for 12 days) (Gao et al., 2024).

### Microspore isolation buffer and culture media preparation

Microspore isolation buffer (MIB) was prepared with 60 g/L of mannitol, 1.1 g/L of CaCl_2_, 20 mg/L of colchicine, and 0.976 g/L of 2-(N-morpholino) ethane sulfonic acid hydrate (MES). Five callus induction media (CIMs) with different basal media and carbon sources (**Table 2**) were evaluated. The plant differentiation medium (DM) was previously described by Guo et al. (2024), and it contained half-strength Murashige and Skoog (1/2MS) as the basal culture medium with 30 g/L maltose, 2.0 mg/L 6-furfurylamino-purine (kinetin, KT), 1.0 mg/L 6-benzylaminopurine (6-BA), 0.5 mg/L naphthalene acetic acid (NAA), and 6.0 g/L agar. The rooting medium (RM) was based on 1/2MS basal medium with 30 g/L sucrose, 0.4 mg/L NAA, 3.0 mg/L paclobutrazol, and 6.0 g/L agar. The pH of all media was adjusted to 5.8. The MIB and CIMs were sterilized using a syringe filter with 0.22-µm polyethersulfone (PES), while the DM and RM were sterilized at 0.11 MPa and 121°C for 20min.

**Table 2:** Different callus induction media (CIMs) used in this study.

CIM	Basal medium	Carbon source	Other additives
I	NB	90 g/L sucrose	0.5 g/L proline, 0.5 g/L glutamine, 0.3 g/L hydrolysed casein, 2.00 mg/L 2,4-D, 0.5 mg/L KT, 0.976 g/L MES
II	NB	90 g/L maltose	
III	M8	90 g/L maltose	
IV	NB	45 g/L sucrose and 45 g/L maltose	
V	N6	90 g/L maltose	

### Microspore isolation and callus induction

Microspores were isolated according to Chen et al. (2023). The rice panicles were surface-sterilized with 10% NaClO for 10min and then rinsed with sterilized water 4–5 times. Anthers with a length reaching from one-third to one-half of the total floret length were separated and transferred into 50-mL tubes, with approximately 900 anthers per tube. Then, 15 mL MIB was added to each tube, and the anthers in the tube were quickly rotary-cut twice using a homogenizer at a speed of 5000 rpm for 2–3 s to release the microspores. The mixture was filtered through a metal sieve with a 100-μm aperture, and the filtrate was centrifuged at 100×*g* for 5min. The supernatant was discarded, and the pellet containing the microspores was resuspended by adding 3 mL MIB and transferred to a Petri dish (6cm in diameter). The Petri dishes were sealed with parafilm and placed in an incubator at 26°C in the dark for 1–2 days. The mixture was then transferred to 50 mL tubes, mixed with 21% maltose solution, and centrifuged at 100×g for 5min. The supernatant containing microspores was selected. Before callus induction, the microspores were washed and suspended in CIM, and the density was adjusted to a concentration of approximately 1.0×10^5^ microspores per mL. Subsequently, 1.0 mL of the microspore suspension was transferred to each Petri dish (30 mm×15 mm) and sealed with parafilm. For callus induction, Petri dishes containing microspores and CIMs were placed in an incubator at 26°C in the dark at 60–75% relative humidity. For most rice genotypes, the calli in each Petri dish were weighed 4 weeks after induction. Q12 and Q13 genotypes were weighed after 10 weeks because of their slower responses to callus induction, and calli of the Q14 genotype were weighed after 8 weeks. Callus induction of Q9 in CIM IV was more complex, and calli were weighed both after 7 and 10 weeks because of their slower and varied responses to callus induction. The callus yields were used to evaluate the capacity for callus induction. After weighing, calli were immediately transferred to DM for plant differentiation.

### Plant regeneration and rooting

Plant regeneration and rooting were performed according to the methods described by Guo et al. (2024). The calli induced in each Petri dish were transferred to 100-mL triangular flasks with 50 mL DM and then placed in an artificial climate room at 24°C, 60–75% relative humidity and 16-h photoperiod for plant differentiation. After 4–5 weeks, regenerated green plantlets more than 2cm in height were transferred to 200 mL jars containing 70 mL RM for seedling enhancement and rooting. All regenerated plantlets (including both green and albino plantlets) were counted to determine the plant regeneration ability. Plant regeneration frequency=total number of regenerated plantlets×100 (mg)/total callus yield (mg); Green plant regeneration frequency=total number of regenerated green plantlets×100 (mg)/total callus yield (mg). The correlation between the callus yield and the number of regenerated green seedlings was analyzed by using the Pearson method.

### Transplanting and ploidy determination

Regenerated seedlings were transplanted at the Lingshui Experimental Base in Lingshui City of Hainan Province of China (Lingshui city). Ploidy status was evaluated during the grain filling stage based on whether normal seed setting occurred, and the plant height and spikelet size were also measured as references (Guo et al., 2024).

### Statistical analysis

Two-way ANOVA of callus yield was performed using the SPSS 26 software. After obtaining a significant result (*P*<0.05, F-test) from the analysis of variance (ANOVA), the analysis assessed the statistical significance of differences in callus yield between media using the least significant difference (LSD) at the 5% (*P*<0.05) level of significance.

## Results

### Effects of the CIM on callus induction among rice genotypes

A major challenge during the isolated microspore culture process is contamination. In this experiment, the overall contamination control was very good, as only four culture tests with two rice genotypes were seriously affected (**Table 3**). Thus, the experiment was suitable for further analysis. The effects of the CIM on callus induction were significantly different (**Table 3**). Calli were successfully induced for eight rice genotypes using CIM I. CIM II was more efficient and resulted in calli for 12 rice genotypes. Although CIM III was used for callus induction of 14 rice genotypes, calli were successfully induced in 12. CIM IV was used for callus induction of 13 rice genotypes, but only 6 formed calli. Experiments were also carried out on 13 rice genotypes using CIM V, 10 of which successfully produced calli. Thus, it was showed that the largest number of rice genotypes could be induced to produce calli by using CIMs II and III, while CIM IV induced the smallest number of rice genotypes to produce calli. Two-way Analysis of Variance (Two-way ANOVA) showed that there were highly significant differences in callus yield among different CIMs and among different rice genotypes (*P*<0.01); in addition, there was also a highly significant interaction between CIMs and rice genotypes on the effect of callus yield (*P*<0.01) (**Supplementary Table S1**). In the multiple comparison analysis of callus yield among different CIMs (**Supplementary Figure S1**), CIM III exhibited the highest callus yield, while CIM I and CIM IV showed the lowest callus yield. Therefore, the CIM III was the best for callus induction in different rice genotypes based on the above results.

**Table 3:** Callus induction using different callus induction media (CIMs) for different rice genotypes (continued).

CIM/Rice genotype		Q1	Q2	Q3	Q4	Q5	Q6	Q7	Q8
I	Callus yield per petri dish (mg)	42.23 ± 6.26a	no callus	no callus	22.86 ± 20.68c	25.45 ± 16.46c	no callus	no callus	9.77 ± 0.15b
	Number of petri dishes	3			15	11			3
II	Callus yield per petri dish (mg)	36.8 ± 11.28a	23.77 ± 6.59b	no callus	102.51 ± 56.25b	86.03 ± 40.02a	35.10	no callus	48.77 ± 8.65b
	Number of petri dishes	5	3		15	4	1		3
III	Callus yield per petri dish (mg)	contaminated	100.20 ± 48.87a	41.07 ± 4.27	156.92 ± 71.12a	74.63 ± 17.35a	no callus	not tested	142.73 ± 80.46a
	Number of petri dishes		3	3	12	7			3
IV	Callus yield per petri dish (mg)	contaminated	no callus	no callus	19.50 ± 9.44c	68.93 ± 10.62a	not tested	not tested	38.50 ± 3.62b
	Number of petri dishes				6	4			3
V	Callus yield per petri dish (mg)	not tested	56.38 ± 30.75ab	no callus	not tested	41.96 ± 16.14b	no callus	no callus	167.42 ± 49.08a
	Number of petri dishes		6			5			12
CIM/Rice Genotype		Q9	Q10	Q11	Q12	Q13	Q14	Q15	
I	Callus yield per petri dish (mg)	35.95 ± 13.05b	no callus	51.58 ± 17.24bc	45.00	no callus	7.60 ± 3.96b	contaminated	
	Number of petri dishes	6		5	1		2		
II	Callus yield per petri dish (mg)	77.03 ± 72.01ab	23.12 ± 20.97b	55.28 ± 15.19b	39.75 ± 14.07a	no callus	32.65 ± 16.89b	80.67 ± 5.44b	
	Number of petri dishes	9	6	6	2		6	3	
III	Callus yield per petri dish (mg)	73.27 ± 8.63ab	122.00 ± 42.27a	108.75 ± 24.00a	70.72 ± 38.00a	151.47 ± 24.40a	76.23 ± 19.33a	116.30 ± 21.27b	
	Number of petri dishes	3	3	8	5	3	3	3	
IV	Callus yield per petri dish (mg)	60.35 ± 45.33ab	no callus	10.73 ± 3.26d	no callus	no callus	36.33 ± 17.36b	contaminated	
	Number of petri dishes	2		3			3		
V	Callus yield per petri dish (mg)	100.70 ± 55.81a	55.16 ± 43.82b	28.25 ± 11.32cd	46.88 ± 16.14a	102.17 ± 33.69a	113.40	196.65 ± 44.76a	
	Number of petri dishes	6	5	4	5	3	1	3	

Different letters after values of “mean ± s.d.” indicate significant differences among different CIMs for each rice genotype, and no letters after the values of “mean ± s.d.” indicate no comparisons because of only one replicate.

Upon comparing the effects of CIMs I, II, and IV, which only differed in their carbon sources, maltose was found to induce the largest number of rice genotypes to produce calli, followed by sucrose, and the combination of two carbon sources resulted in the smallest number of rice genotypes. Therefore, it was recommended to use maltose as the single carbon source in CIM.

Moreover, CIM III exhibited the best callus induction according to the multiple comparison of callus yield. Therefore, it was recommended to select CIM III with M8 basal medium for callus induction.

### Effects of calli derived from different CIMs on plant differentiation of different rice genotypes

Rice genotype Q7 failed to induce calli on any CIM, preventing further differentiation. As the same differentiation media were used for all calli, differences in regenerated plantlets during the differentiation process were caused by variations in rice genotypes and callus origins (from different CIMs). As shown in **Table 4**, among the eight rice genotypes that successfully produced calli on CIM I, only four regenerated green plantlets. On CIM II, 7 of 12 rice genotypes produced green plantlets, with one genotype yielding only albino plantlets. On CIM III, 9 of 12 rice genotypes regenerated green plantlets, and one produced albino plantlets. On CIM IV, 4 of 6 rice genotypes produced green plantlets, and only one had albino plantlets. On CIM V, 9 of 10 rice genotypes successfully regenerated green plantlets.

**Table 4:** Plant regeneration using different callus induction media (CIMs) for different rice genotypes.

CIM/Rice genotype		Q1	Q2	Q3	Q4	Q5	Q6	Q8	Q9	Q10	Q11	Q12	Q13	Q14	Q15
I	Total callus yield (mg)	126.7	not tested	not tested	342.9	279.9	not tested	29.3	215.7	not tested	257.9	45.0	not tested	15.2	not tested
	Number of green plantlets	4			58	18		no plant	16		no plant	no plant		no plant	
	Number of albino plantlets	4			70	29			0						
	Plant regeneration frequency	6.31			37.33	16.79			7.42						
	Green plant regeneration frequency	3.16			16.91	6.43			7.42						
II	Total callus yield (mg)	184.0	71.3	not tested	1537.6	344.1	35.1	146.3	693.3	138.7	331.7	79.5	not tested	195.9	242.0
	Number of green plantlets	8	0		238	99	no plant	no plant	164	2	no plant	no plant		9	24
	Number of albino plantlets	21	5		227	54			104	3				3	23
	Plant regeneration frequency	15.76	7.01		30.24	44.46			38.66	3.60				6.13	19.42
	Green plant regeneration frequency	4.35	0		15.48	28.77			23.65	1.44				4.59	9.92
III	Total callus yield (mg)	not tested	300.6	123.2	1883.0	522.4	not tested	428.2	219.8	366.0	870.0	363.6	454.4	228.7	348.9
	Number of green plantlets		no plant	no plant	259	134		0	15	19	2	5	5	21	6
	Number of albino plantlets				158	41		1	1	17	0	0	3	0	4
	Plant regeneration frequency				22.15	33.50		0.23	7.28	9.84	0.23	1.38	1.76	9.18	2.87
	Green plant regeneration frequency				13.75	25.65		0	6.82	5.19	0.23	1.38	1.10	9.18	1.72
IV	Total callus yield (mg)	not tested	not tested	not tested	117.0	275.7	not tested	115.5	120.7	not tested	32.2	not tested	not tested	109.0	not tested
	Number of green plantlets				5	15		no plant	35		0			3	
	Number of albino plantlets				34	20			7		2			0	
	Plant regeneration frequency				33.33	12.69			34.80		6.21			2.75	
	Green plant regeneration frequency				4.27	5.44			29.00		0			2.75	
V	Total callus yield (mg)	not tested	338.3	not tested	not tested	209.8	not tested	2009.0	604.2	275.8	113.0	234.4	306.5	113.4	393.3
	Number of green plantlets		1			40		1	36	6	no plant	6	2	36	5
	Number of albino plantlets		2			39		19	18	21		2	0	2	4
	Plant regeneration frequency		0.89			37.65		1.00	8.94	9.79		3.41	0.65	33.51	2.29
	Green plant regeneration frequency		0.30			19.07		0.05	5.96	2.18		2.56	0.65	31.75	1.27

Plant regeneration frequency is the number of plantlets per 100 mg of calli, and green plant regeneration frequency is the number of green plantlets per 100 mg of calli.

In conclusion, CIMs III and V showed the best performance based on the number of rice genotypes that regenerated green plantlets from induced calli, followed by CIM II, whereas CIMs I and IV resulted in fewer plantlets. Additionally, CIMs II, III, and V ensured green plantlet regeneration for all 12 *japonica* rice genotypes, whereas the two *indica* rice genotypes failed. These three CIMs used maltose as an additional carbon source, differing only in basal media. The results indicate that maltose as an additional carbon source promoted callus induction among rice genotypes and facilitated subsequent differentiation.

### Ploidy identification in regenerated plants of different rice genotypes

To obtain more regenerated rice plantlets for ploidy identification, the differentiation was continued after counting the regenerated plantlets mentioned above, resulting in a higher final number of regenerated green plantlets. Since *indica* rice genotypes Q3, Q6, and Q7 failed to produce green plantlets, ploidy identification was performed on the remaining 12 *japonica* rice genotypes. Except for Q8 and Q11, approximately 50 regenerated seedlings per genotype were randomly selected for analysis. The diploid proportion ranged from 14.3 to 98%, with eight genotypes exceeding 60% and four genotypes exceeding 90% (**Table 5**). This confirms that isolated microspore culture achieved a high diploid ratio, indicating that, in most cases, a high proportion of DH plants can be obtained without artificial chromosome doubling.

**Table 5:** Ploidy identification of regenerated plants of different rice genotypes.

Rice genotype	Total number of transplanted seedlings	Number of haploid plants	Number of diploid plants	Percentage of haploid plants (%)	Percentage of diploid plants (%)
Q1	54	4	50	7.4	92.6
Q2	52	16	36	30.8	69.2
Q4	50	3	47	6.0	94.0
Q5	50	11	39	22.0	78.0
Q8	27	18	9	66.7	33.3
Q9	50	14	36	28.0	72.0
Q10	49	1	48	2.0	98.0
Q11	21	18	3	85.7	14.3
Q12	50	6	44	12.0	88.0
Q13	53	31	22	58.5	41.5
Q14	51	29	22	56.9	43.1
Q15	50	2	48	4.0	96.0

## Discussion

**Figure 2:**
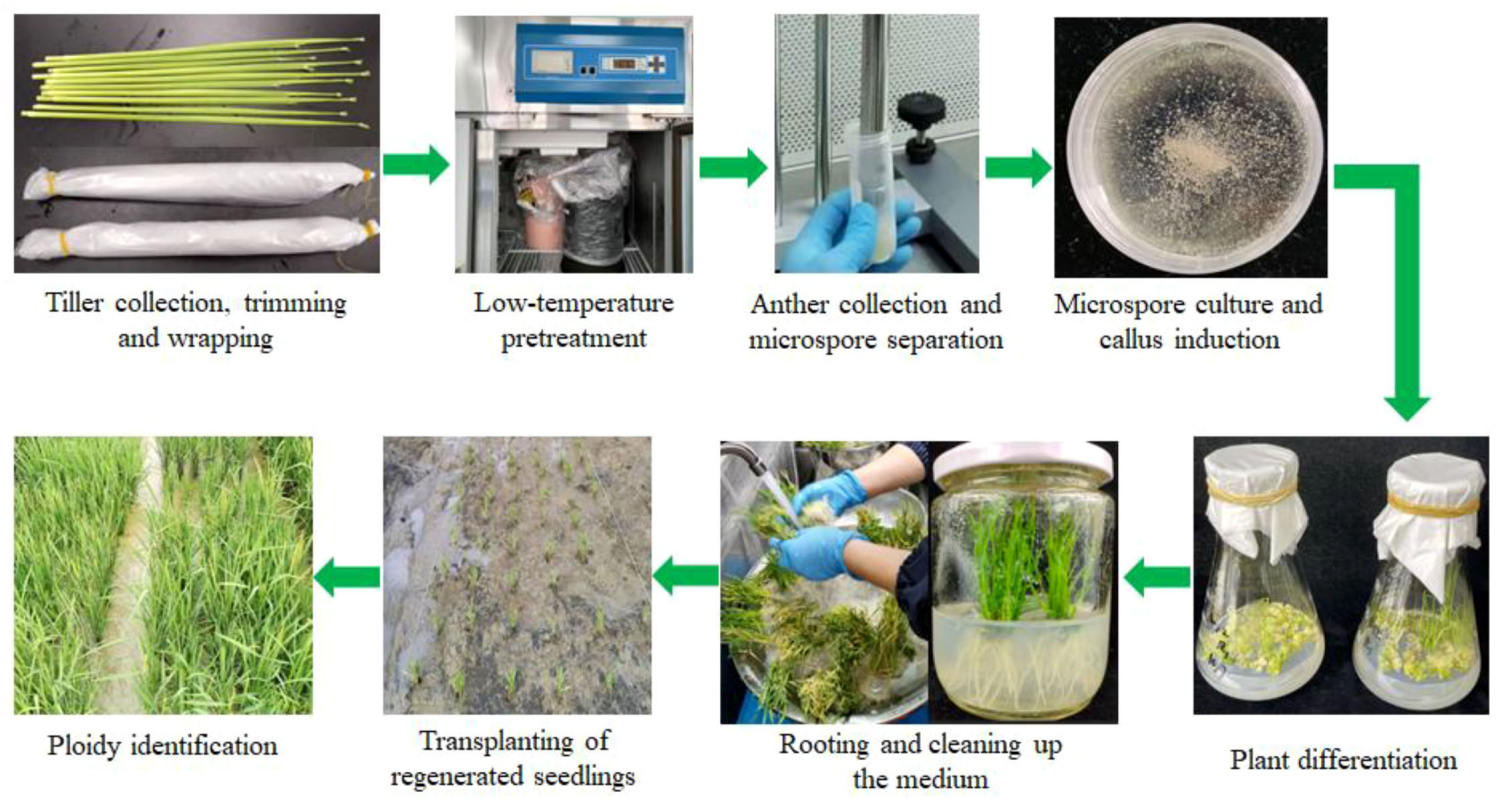
The workflow of isolated microspore culture in rice.

The establishment and optimization of the isolated microspore culture method in rice often face challenges, such as contamination during operation, difficulty in controlling microspore viability, and poor universality of the culture system. In this study, the isolated microspore culture method was optimized for 15 rice genotypes, including the main cultivated varieties in the Yangtze River Delta region of China and core parental lines used in Shanghai Academy of Agricultural Sciences and model varieties, providing a solid basis for the development of doubled haploid breeding in this region (**Figure 2**).

For tiller collection and anther selection, the standards for anther culture established by Guo et al. (2024) were also used for isolated microspore culture in this study because of operational convenience. In terms of pretreatment, the previous study has shown that the tillers required more than 10 days of low-temperature treatment to efficiently activate a microspore response, and there is a positive correlation between the treatment duration and induction efficiency (Gao et al., 2024). However, excessive treatment causes damage and decay in tiller tissues. After balancing the treatment effects and tissue tolerance, the low-temperature pretreatment duration in this study was set to 12 days. In contrast, Rahman et al. (2022) found that 7 days of low-temperature treatment was sufficient. The microspore isolation method from anthers adopted here used a homogenizer, and it has been shown to achieve efficient microspore separation and callus induction and significantly reduce the number of albino seedlings (Islam et al., 2013).

Callus induction is a core step in isolated microspore culture of rice; thus, this step was optimized in this study. Comparing the basal media, there were obvious differences in their applicability among rice genotypes. CIM III, which used M8 (callus induction medium for cereals) basal media, exhibited higher callus induction efficiency. Regarding additional carbon source selection, previous studies have shown significant discrepancies in additional carbon sources. Zapata et al. (1991); Ogawa et al. (1994), and Rahman et al. (2022) favored sucrose, whereas Raina and Irfan (1998) showed that maltose was more advantageous. Gao et al. (2024) reported the comparable effects of both. Using multi-genotypic testing, this study validated that maltose performed optimally during callus induction. Considering other supplements, glutamine and proline have been reported to promote callus induction and plant regeneration (Zapata et al., 1991). These amino acids were also added to CIMs in this study. After comprehensive comparison of divergent reports by Rahman et al. (2022); Islam et al. (2013), and Gao et al. (2024), a combination of 0.5 mg/L cytokinin and 2.0 mg/L 2,4-dichlorophenoxyacetic acid was used to optimize plant hormones and their ratios. This combination demonstrated optimal callus induction efficiency among the genotypes evaluated in this study.

For plant differentiation, no regenerated seedlings were obtained for *indica* rice genotypes. Therefore, we conducted a Pearson correlation analysis on the callus yield and the number of regenerated green seedlings produced using CIMs II and III, which both induced calli in the most rice genotypes. Both showed highly significant positive correlations (*P*<0.01), with correlation coefficients of 0.935 and 0.841, respectively. This suggests that the absence of regenerated green seedlings in *indica* rice genotypes might be related to their lower callus yields. The culture scale of *indica* rice genotypes could be expanded to obtain regenerated green seedlings, or callus induction could be further improved. It was found that the callus induced later from these two *indica* rice genotypes (Q3 and Q6) were more likely to differentiate into green seedlings, but further exploration was needed for revealing the underlying mechanisms.

For ploidy identification, except for Q11 and Q13, the spontaneous chromosome doubling rate of regenerated seedlings from isolated microspore culture was higher than that previously observed in those from anther culture (Guo et al., 2024). Approximately one-third of the rice genotypes had a spontaneous chromosome doubling rate exceeding 90%. Compared to anther culture, colchicine, which is typically used to trigger microspore reprogramming, was added to MIB. However, the correlation between colchicine addition to MIB and the high spontaneous chromosome doubling rate of regenerated seedlings requires further investigation, which will be an important future direction for studies on chromosome doubling.

## Conclusion

In summary, an isolated microspore culture method, suitable for different rice genotypes, was established. The callus induction medium was optimized in this study, and the effect of adding maltose was better. There was no need for artificial chromosome doubling and seedling nursery, and the regenerated seedlings could be directly transplanted into the field after rooting and strengthening, which simplified the procedure and was conducive to large-scale application. At the same time, the spontaneous chromosome doubling rate of regenerated seedlings in isolated microspore culture was obviously higher than that in anther culture, which could better meet the breeding needs. However, the regeneration of green seedlings was still difficult, and the next step would focus on optimizing this step.

## Data Availability

The original contributions presented in the study are included in the article/**Supplementary Material**. Further inquiries can be directed to the corresponding authors.

## Supplementary material

Supplementary Table 1 Two-way ANOVA of callus yield in different rice genotypes and different callus induction media.

Supplementary Figure 1 Callus yields that were induced by using different callus induction media (CIMs). Means and standard deviations are shown, and different letters indicate significant differences (P < 0.05).
