# Supplementary material for: Workflow for efficiently isolating microspore cultures of different rice genotypes by optimizing the callus induction medium
Source: Front Plant Sci. 2025 Sep 30;16:1662463. doi: 10.3389/fpls.2025.1662463 (PMC12518337; doi:10.3389/fpls.2025.1662463)
Supplement: Supplementary Table 1 — Two-way ANOVA of callus yield in different rice genotypes and different callus induction media. [file Table1.docx]

Table S1. Two-way ANOVA of callus yield in different rice genotypes and different callus induction media

| Source | SS | df | MS | F | Sig. |
| --- | --- | --- | --- | --- | --- |
| Callus induction medium | 112411.16 | 4.00 | 28102.79 | 19.95 | 0.00 |
| Rice genotype | 72178.05 | 13.00 | 5552.16 | 3.94 | 0.00 |
| Callus induction medium ×Rice genotype | 151467.54 | 30.00 | 5048.92 | 3.58 | 0.00 |
| Total | 1968053.05 | 235.00 |  |  |  |
