# Supplementary material for: Workflow for efficiently isolating microspore cultures of different rice genotypes by optimizing the callus induction medium
Source: Front Plant Sci. 2025 Sep 30;16:1662463. doi: 10.3389/fpls.2025.1662463 (PMC12518337; doi:10.3389/fpls.2025.1662463)
Supplement: Supplementary Figure 1 — Callus yields that were induced by using different callus induction media (CIMs). Means and standard deviations are shown, and different letters indicate significant differences (P < 0.05). [file DataSheet1.pdf]

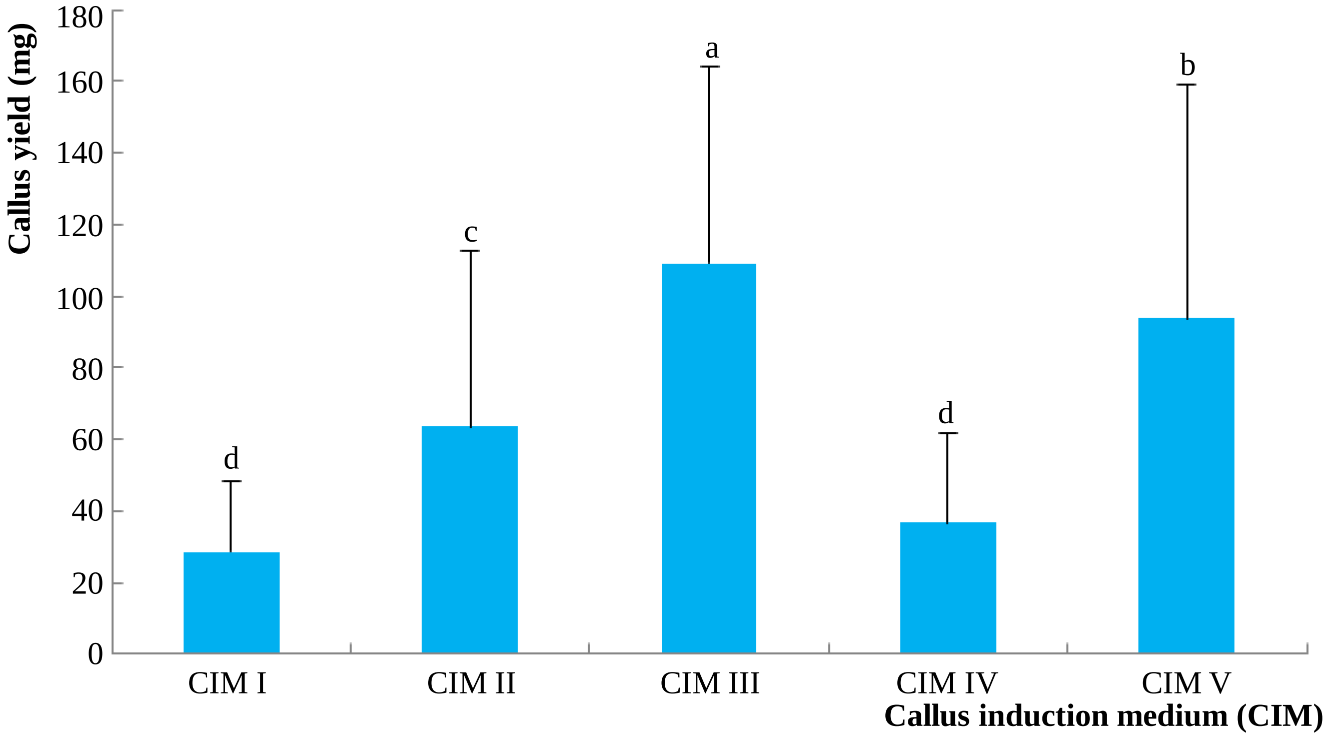

Figure S1. Callus yields that were induced by using different callus induction media (CIMs). Means and standard deviations are shown, and different letters indicate significant differences (*P* < 0.05).
